# Reassessing the validity of using weighted linear models to implement multi-generational GWAS-by-subtraction: a response to Evans et al.

**DOI:** 10.1186/s13104-025-07327-8

**Published:** 2025-06-26

**Authors:** Benjamin Woolf, Dipender Gill, Marcus Munafò, Stephen Burgess

**Affiliations:** 1 MRC Integrative Epidemiology Unit, University of Bristol, Bristol, UK; 2 School of Psychological Science, University of Bristol, Bristol, UK; 3 MRC Biostatistics Unit, University of Cambridge, Cambridge, UK; 4 Department of Epidemiology and Biostatistics, School of Public Health, Imperial College London, London, UK; 5 British Heart Foundation Cardiovascular Epidemiology Unit, Department of Public Health and Primary Care, University of Cambridge, Cambridge, UK

## Abstract

Evans and colleagues for providing a critique on our article “Deriving GWAS summary estimates for paternal smoking in UK biobank: a GWAS by subtraction”. They highlight important limitations in our approach, which we overlooked. They argue that our approach works neither in theory nor in practice– that there are both flaws in the method and in our execution of the method. Here we explore these issues and address what we consider to be the most important criticisms.

**Supplementary Information:**

The online version contains supplementary material available at 10.1186/s13104-025-07327-8.

We would like to thank Evans and colleagues for providing a critique on our article “Deriving GWAS summary estimates for paternal smoking in UK biobank: a GWAS by subtraction” [1, 2]. They highlight important limitations in our approach, which we overlooked. They argue that our approach works neither in theory nor in practice– that there are both flaws in the method and in our execution of the method. Here we explore these issues and address what we consider to be the most important criticisms. We also would like to take this opportunity to apologise for our oversight in not making more explicit the connection to the original GWAS-by-subtraction by Demange and colleagues that inspired our title and original estimation Eq. (3).

## Entering model land

**Fig. 1:**
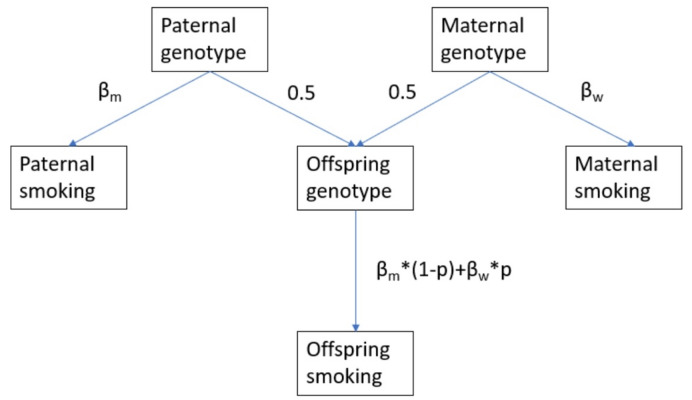
Path diagram illustrating the assumptions of our proposed GWAS-by-subtraction. β_m_ is the association among men, β_w_ is the association among women, p is the proportion of women in the offspring GWAS. Thus, our GWAS assumes that: (1) the paternal genotype– parental smoking associations are the same as the offspring genotype - offspring smoking associations in a male only offspring sample (i.e. generational homogeneity), and (2) sex-combined estimates are a weighted average of female and male specific effects. The weights in our updated formula are 2*p and 2*(1-p) because offspring genotype– parental phenotype associations are half the parental genotype– parental phenotype associations. β_w_ is therefore twice the offspring genotype– maternal phenotype association

Evans and colleagues clarify implicit assumptions about homogeneity across generations and sexes. Our original Eq. (1) can be updated to account for different sex-specific effects. The beta for an autosomal SNP in a sex-combined GWAS (i.e., a GWAS including both men and women) is the average of the sex-specific effects weighed by the proportion of men and women. Given our original Eqs. (4) and (5), that the GWASs are sampled from the same target population, and generational homogeneity, we can update Eq. (6) to:

$$\:{\widehat{\beta\:}}_{CG\_FP}=\:\:\frac{{\widehat{\beta\:}}_{CG\_CP}-\:{w}_{1}*{\widehat{\beta\:}}_{CG\_MP}}{{w}_{2}}$$

where $\:{w}_{1}$ is the twice the proportion of females and $\:{w}_{2}$ is twice the proportion of males in the offspring generation GWAS (see Fig. 1), $\:{\widehat{\beta\:}}_{CG\_FP}$ is the estimated association between the child’s genotype and the paternal phenotype, $\:{\widehat{\beta\:}}_{CG\_MP}$ is the estimated association between the child’s genotype and the maternal phenotype, and $\:{\widehat{\beta\:}}_{CG\_CP}$ is the estimated association between the child’s genotype and the child’s phenotype. Generational homogeneity requires that the mother’s variant-phenotype association is the same as her daughter’s variant-phenotype association, and the father’s variant-phenotype association is the same as his son’s variant-phenotype association. For a sample with a 50:50 sex ratio, this equation reduces to our original Eq. (6).

Evans and colleagues also criticize our estimator for being inefficient and having a conservative standard error. While our new estimator might be less efficient than their suggested one, it does allow for sex effect modification. We agree that our original standard error is conservative, but using a conservative standard error is not necessarily a problem. However, we can update the standard error to incorporate any $\:{\widehat{\beta\:}}_{CG\_MP}$ and $\:{\widehat{\beta\:}}_{CG\_CP}$ covariance:

$$\begin{array}{*{20}{l}}{\:se\left( {{{\widehat {\beta \:}}_{CG\_FP}}} \right) = }\\{\:\:\sqrt {\frac{\begin{array}{l}var\left( {{{\widehat {\beta \:}}_{CG\_CP}}} \right) + {w_1}^2*var\left( {{{\widehat {\beta \:}}_{CG\_MP}}} \right)\\- 2{w_1}*cov({\widehat {\beta \:}_{CG\_CP}},{\widehat {\beta \:}_{CG\_MP}})\end{array}}{{{w_2}^2}}} }\end{array}$$

where the covariance can be estimated using LD score regression, as suggested by Evans and colleagues (and others) [1, 3].

## Exploring the subtraction

The subtraction formula above assumes there are no systematic errors in the GWAS summary statistics. While we noted sources of error such as measurement error and residual confounding in our original article, we failed to highlight that we are assuming that *all* differences are not due to *any* form of error [3]. This is implausible, and may account for the inflation seen in the original QQ plot.

The Neale group provide both sex-combined and sex-stratified GWAS summary statistics using UK Biobank [4]. As a control analysis, we compared the actual male estimates to male estimates derived by subtracting the female estimates from the combined-sex estimates, using the formula above but where $\:{w}_{1}$and $\:{w}_{2}$ are the proportion of females and males respectively and the betas are female-specific or sex-combined betas for the same generation.

The results of this analysis are presented in Table 1. The median differences between the estimates from the actual GWAS among men and the estimates approximated by subtracting the female effects from the sex-combined GWAS, with associated interquartile ranges, are centred on zero. While the estimates of genomic inflation do differ [5], this appears to be due to differences in the size of the standard errors. The associated QQ plots found in Supplementary Figure S1 present a similar story. This reassures us that the subtraction method can on average provide unbiased estimates.

**Table 1:** Comparison of actual male-only GWASs with male GWAS-by-subtraction estimates

Trait	Median difference in beta values (IQR)*	Median difference in SE (IQR)*	LD Score regression intercept	
			Male GWAS	Male GWAS-by-subtraction
Height^1^	0.000 (-0.003 to 0.004)	-0.005 (− 0.015 to– 0.003)	1.144	6.649
Weight^1^	0.000 (-0.002 to 0.002)	-0.003 (-0.007 to -0.001)	1.043	1.446
Hyper-tension^2^	0.000 (0.000 to 0.000)	0.000 (-0.001 to 0.000)	1.039	1.195
Birth weight^1^	0.000 (-0.001 to 0.001)	-0.002 (-0.006 to -0.001)	1.021	1.200
Current Smoking^2^	0.000 (0.000 to 0.000)	0.000 (0.000 to 0.000)	1.016	1.033

* Negative values mean that the GWAS-by-subtraction average values are smaller than the actual GWAS average values

^1^ Units are difference in inverse normalised rank. ^2^ Units are risk differences

In an ideal world we would have access to (and use) summary statistics that directly measure associations with paternal smoking in a large sample. Although our proposed method may in theory offer a quick-and-dirty alternative, we now re-assess whether our application to paternal smoking was valid.

## “In theory, there is no difference between theory and practice. In practice, there is.” Yogi Berra

**Fig. 2:**
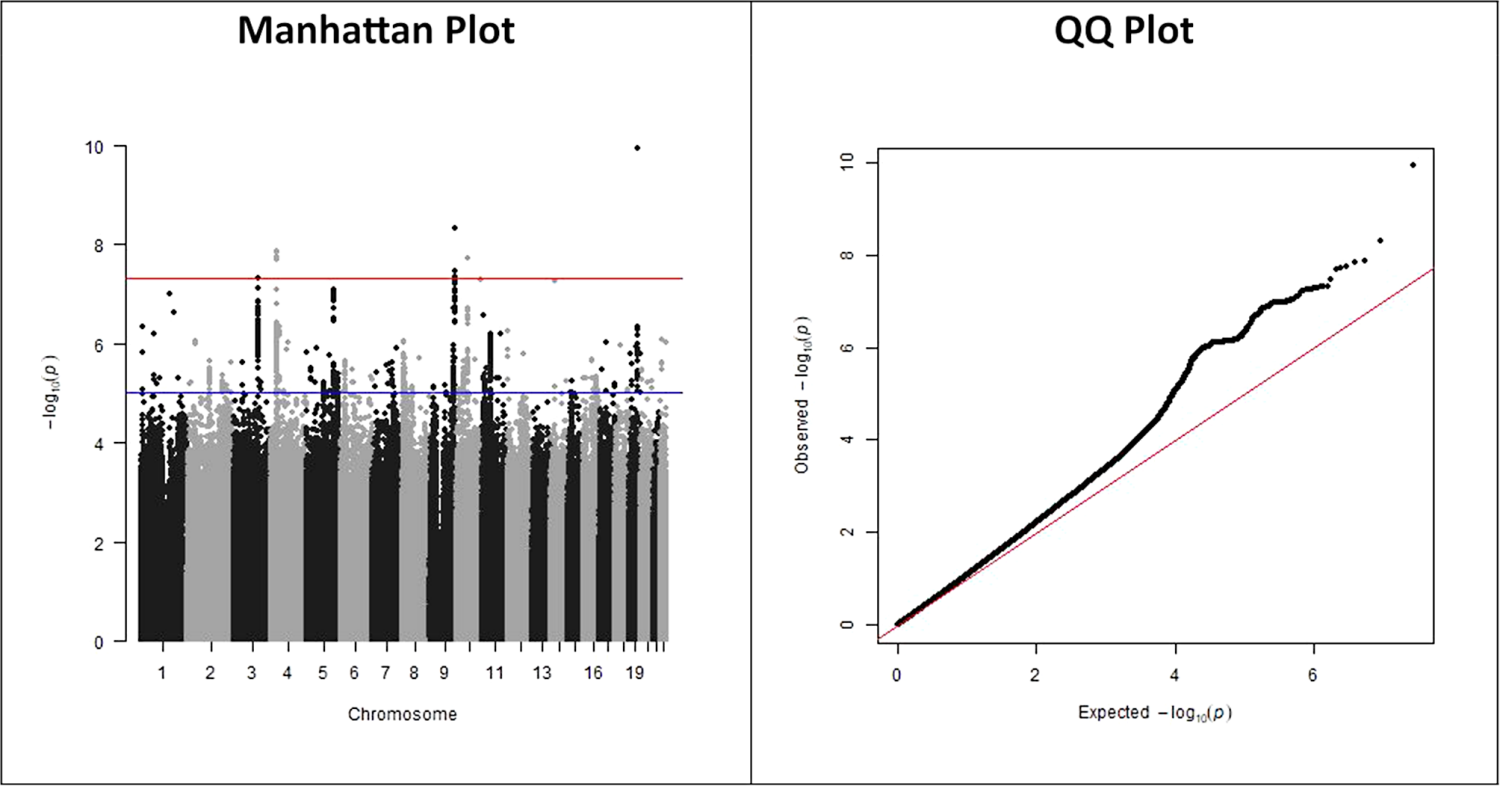
Results for an updated GWAS-by-subtraction for current smoking status. As with our original GWAS-by-subtraction, the units of the two input GWASs were standardised prior to running the GWAS. Specifically, we converted log odds ratios to standardised mean differences by dividing them by $\:\frac{\pi\:}{\sqrt{3}}$ [13]. Where necessary, we converted regression betas into log odds ratios by dividing them by $\:<Emphasis Type="Italic">p</Emphasis>*(1-p)$, where p is the prevalence of smoking in the GWAS [14]. The measures of genomic inflation imply minimal additional inflation (LDSC intercept for GWAS-by-subtraction = 1.015) over that for current or maternal smoking (LDSC intercept = 1.015 and 1.028 respectively). The less extreme genomic inflation in this GWAS-by-subtraction, compared to the original paternal smoking GWAS-by-subtraction, is consistent with these input GWASs being more comparable than those used in the original implementation. Plots were created using the qqman R package [15, 16]

The Neale group analysis above used GWASs derived using identical methods, measures, and target populations. Evans and colleagues are correct to criticize us for combining GWAS with different smoking measures. A more analogous offspring smoking phenotype is current smoking. Like maternal smoking at birth, this is a binary question, asking whether someone smokes at a specific time. In our updated GWAS-by-subtraction (Fig. 2), we used the Neale GWAS of maternal smoking (UK Biobank variable ID: 1787) at birth as a source of $\:{\widehat{\beta\:}}_{CG\_MP}$ estimates, GWAS of current participant smoking (UK Biobank variable ID: 20116) as a source of $\:{\widehat{\beta\:}}_{CG\_CP}$ estimates, and $\:\frac{\text{194,174}}{\text{361,194}\:}\:$as the proportion of women. Using Neale GWASs for both phenotypes ensures that they are methodologically identical.

The generational homogeneity assumption in this instance is that: (1) variant-smoking associations do not differ between the age at which the participant’s mothers gave birth and the participant’s age at recruitment into UK Biobank, (2) age-standardised variant-smoking associations do not differ between the maternal and offspring cohorts, and (3) no measurement error is induced by focusing on maternal smoking at birth rather than at another point in time.

Given the age of UK Biobank participants, most were born before mothers were advised to stop smoking during pregnancy, the third assumption is plausible [6, 7]. The first two assumptions have been made to integrate multigenerational data for various smoking-related phenotypes like lung cancer, lifespan, and birth weight [8–10]. However, the prevalence of smoking is impacted by age and cohort effects [11]. These differences are likely driven by different factors influencing smoking initiation, duration, and so on. As such, there are plausible reasons why genetic associations may be different for different ages and cohorts, similar to recent observations on how the genetics of coffee consumption varies geographically [12]. Thus, although generational homogeneity is not completely implausible, it might not be plausible for smoking. Without access to the type of multigenerational genetic data that would make this method redundant, the acceptability of this assumption is a judgment call and a limitation to be recognised.

## Conclusion

We agree with Evans and colleagues that the summary statistics presented in our original paper should not be used for downstream applications, as the parental and offspring input data were too different. However, we argue that the GWAS-by-subtraction method can still provide unbiased estimates if the two input GWAS sources are similar. The method is likely to be useful in situations where associations of interest are not directly measured, but related associations are available. If the method is used to estimate genetic associations, then these estimates rely on the generational homogeneity assumption. If the method is simply used to identify variants that are more strongly associated in one generation than another, then the validity of these parametric assumptions is less crucial. We would therefore be cautious about the overinterpretation of effect estimates from this approach, and would recommend that usage of the method is restricted to the identification of relevant variants in cases where transgenerational data are not available.

## Electronic supplementary material

Below is the link to the electronic supplementary material.

Supplementary Material 1

## Data Availability

The data used in the analyses is accessible from http://www.nealelab.is/uk-biobank. The code used in the in the analysis is available at 10.17605/OSF.IO/NXGVD#.
