# Supplementary material for: Reassessing the validity of using weighted linear models to implement multi-generational GWAS-by-subtraction: a response to Evans et al
Source: BMC Res Notes. 2025 Jun 26;18:248. doi: 10.1186/s13104-025-07327-8 (PMC12203723; doi:10.1186/s13104-025-07327-8)
Supplement: Supplementary file 1 — Supplementary Material 1 [file 13104_2025_7327_MOESM1_ESM.docx]

Supplementary Figure S1: Comparison of QQ plots for actual male-only GWASs and male GWAS-by-subtraction.

|  | Direct male GWAS | Male GWAS by subtraction |
| --- | --- | --- |
| Height | 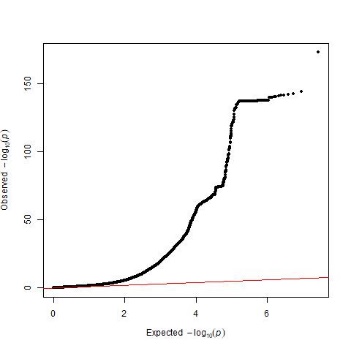 | 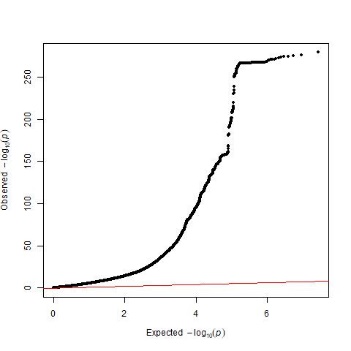 |
| Weight | 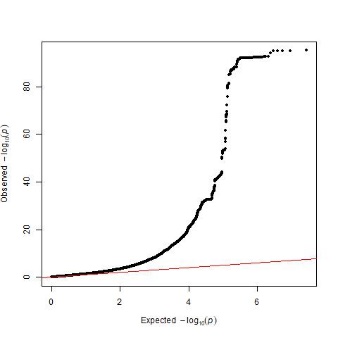 | 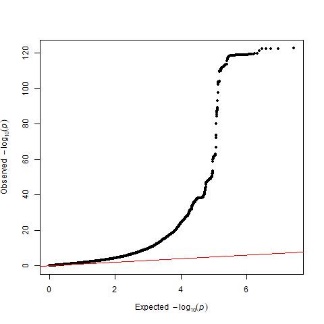 |
| Hypertension | 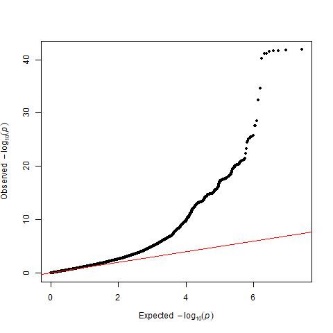 | 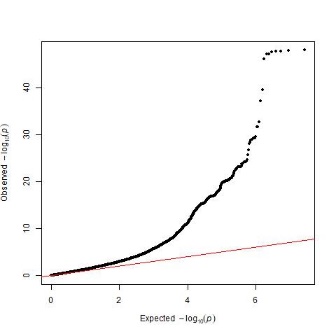 |
| Birth weight | 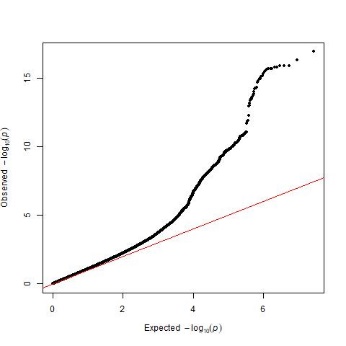 | 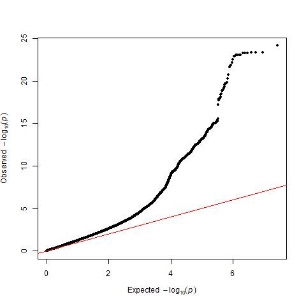 |
| Current Smoking | 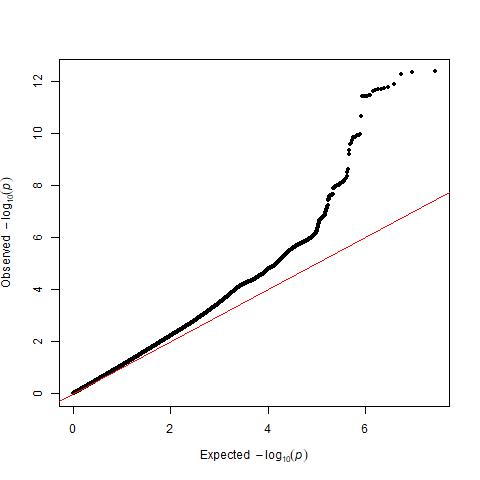 | 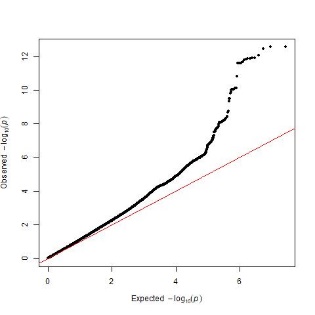 |

Note that the y-axes for each QQ plot are not necessarily the same as the y-axis of other QQ plots in the same row.
